# Breast cancer incidence after the start of mammography screening in Denmark

**DOI:** 10.1038/sj.bjc.6600712

**Published:** 2003-02-10

**Authors:** A H Olsen, A Jensen, S H Njor, E Villadsen, W Schwartz, I Vejborg, E Lynge

**Affiliations:** 1 Institute of Public Health, University of Copenhagen, Blegdamsvej 3, DK-2200 Copenhagen N, Denmark; 2 Centre of Diagnostic Imaging, University Hospital Copenhagen, Blegdamsvej 9, DK-2100 Copenhagen Ø, Denmark; 3 Mammography Screening Clinic, University Hospital Odense, Kløvervænget 10, DK-5000 Odense, Denmark

**Keywords:** malignant neoplasms, breast, mammography

## Abstract

Mammography screening may lead to overdiagnosis of asymptomatic breast cancers, that would otherwise not have given rise to clinical symptoms. This aspect was studied in three regional screening programmes in Denmark, which started in Copenhagen municipality, Fyn county, and Frederiksberg municipality in 1991, 1993, and 1994, respectively. In these regions, we compared time trends in incidence of invasive breast cancer with the rest of Denmark. Since the number of clinical mammograms was relatively low, it was reasonable to assume that the breast cancer incidence outside the three screening regions represented the incidence of a population with low-intensity opportunistic screening. In Copenhagen and Fyn, a prevalence peak in incidence was seen during the first invitation round. During the subsequent invitation rounds, the incidence dropped to a level in line with the incidence expected without screening. The pattern was different in the small municipality of Frederiksberg, where the sensitivity was low during the first invitation round. Inclusion of screen-detected ductal carcinoma *in situ* cases did not change these results. The experiences from Copenhagen and Fyn show that organised mammography screening can operate without overdiagnosis of breast cancer.

An overview of the five Swedish trials found that after 9 years of follow-up, screening reduced breast cancer mortality by 29% in women aged 50–69 at the time of screening (Nyström *et al*, 1993). Based on these results, mammography screening has disseminated broadly both in organised programmes as in the United Kingdom and the Netherlands, and as opportunistic screening.

Mammography screening of asymptomatic women may also have potential negative side effects such as overdiagnosis of asymptomatic breast cancers that would otherwise not have given rise to clinical symptoms. Overdiagnosis is difficult to study. The introduction of screening will lead to a temporary increase in the incidence of breast cancer as prevalent cases are picked up by the screening. It may therefore be difficult to distinguish between the expected ‘prevalence peak’ and an increase in incidence derived from overdiagnosis. This is particularly the case if screening is introduced gradually.

In Denmark, organised mammography screening is offered to 50–69-year-old women every second year in only three out of 16 administrative regions, and very little opportunistic screening takes place in the remaining regions. Breast cancer incidence can be monitored through the Danish Cancer Register. Denmark is therefore a good ‘laboratory’ for studying the effect of organised mammography screening on breast cancer incidence. We report here on the incidence trends in the three Danish mammography screening regions and in the rest of Denmark.

## MATERIALS AND METHODS

### Screening programmes

The Copenhagen programme started on 1 April 1991 targeting all 40 000 women aged 50–69 at the start of an invitation round. The Fyn programme started in November 1993 targeting all 50 000 women aged 50–69 on the day of invitation. The first invitation round took place from November 1993 and 31 December 1995, but the activity was low during the first 2 months, and subsequent invitation rounds covered exactly 2 years starting from the 1st of January. The Frederiksberg programme started in June 1994 covering 10 000 women defined as in Copenhagen, and in 1996 was incorporated into the Copenhagen programme. The three programmes together covered 100 000 women, equivalent to 20% of Danish women aged 50–69.

### Mammograms

We distinguished between screening mammography offered in the organised programmes to asymptomatic women, and clinical mammography used for symptomatic women and sometimes also for opportunistic screening of asymptomatic women. Our counting unit is a mammographic examination, which will normally include examination of both breasts, here termed a mammogram.

The numbers of screening mammograms were retrieved from the database in the Copenhagen Hospital Organisation, covering both Copenhagen and Frederiksberg (Mammography Screening Evaluation Group, 1998), and from the health care database for Fyn (Sundhedsstyrelsen, 1997).

The numbers of clinical mammograms performed in 1983–1987 were found in publications from the National Board of Health (Sundhedsstyrelsen, 1989, 1995,^1997^). For 1990–1991, data were available from a survey in selected counties (Andreasen *et al*, 1994), and the total number for Denmark was estimated on this basis. We estimated the proportion of clinical mammograms used by women aged 50–69 from surveys conducted in 1984 (Pociot *et al*, 1986) and 1990–1991 (Andreasen *et al*, 1994). For 2000, individual records on clinical mammograms were collected directly from all radiology centres.

### Incidence of invasive breast cancer

The numbers of invasive breast cancers from 1985 to 1997 were retrieved from the Danish Cancer Register, ICD-7 code 170 (International Classification of Diseases and Causes of Death 7th edition), and behaviour code 3 (Sundhedsstyrelsen, 2001). To calculate incidence rates, corresponding population data were retrieved from Statistics Denmark (http://www.statistikbanken.dk). Invasive breast cancers and population data were tabulated for 2-year periods for the target groups of the screening programmes. For each of the three regions, similar populations were defined for the periods before screening started, and for a comparison group including the rest of Denmark except Copenhagen, Frederiksberg, and Fyn. Cumulative rates were calculated by adding the age-specific rates for women aged 50–54, 55–59, 60–64, and 65–69 years and multiplying by five. This was then converted to a cumulative risk.

## RESULTS

### Numbers of mammograms

**Figure 1:**
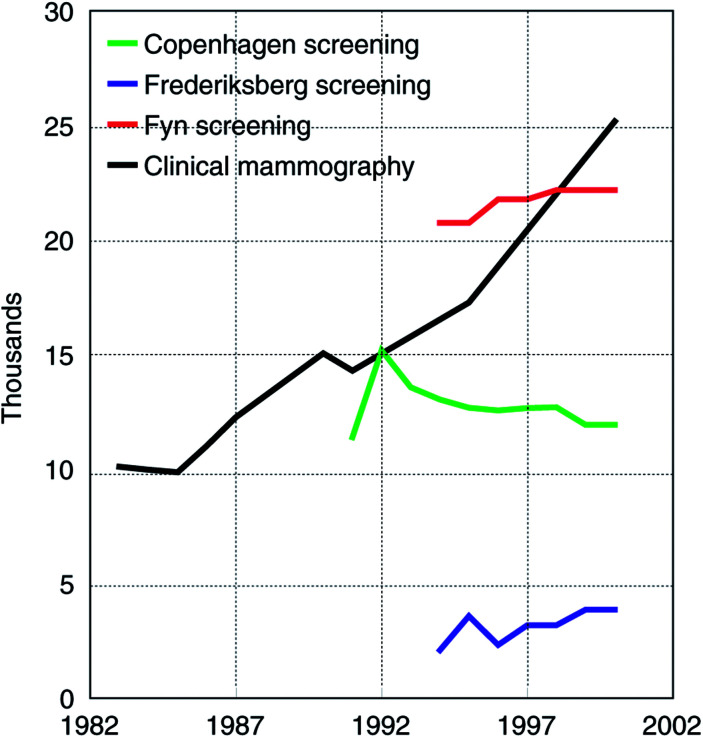
Number of mammography examinations in Denmark in women aged 50–69 during the years 1983–2000.

In total, 97 000 mammograms were performed in Denmark in 2000: 38 000 screening mammograms and 59 000 clinical mammograms. The number of screening mammograms has been stable over time since the programmes started. The number of clinical mammograms increased from about 36 000 per year in 1983–1985, to 40 000 in 1986, 45 000 in 1987–1991, 48 000 in 1995, and 59 000 in 2000. The proportion of mammograms undertaken in women aged 50–69 was close to 30% in both 1984 and 1990–1991. This percentage had, however, increased to 43% in 2000. By linear interpolation we estimated the proportion to be 36% in 1995. The estimated number of clinical mammograms performed in women aged 50–69 thus increased from 10 000 in 1983 to 25 000 in 2000 (Figure 1

).

### Incidence of invasive breast cancer

Figure 2

**Figure 2:**
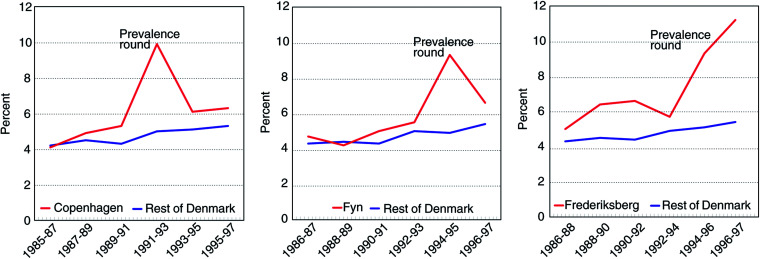
Cumulative risk (in per cent) of incident invasive breast cancer for women aged 50–69 in the three regions with organised mammography screening and in Denmark excluding the screening regions. The pattern in Frederiksberg probably reflects low sensitivity during the first invitation round.

shows the time trends in cumulative risk of invasive breast cancer for women aged 50–69. There was a pronounced prevalence peak during the first invitation round in Copenhagen, after which the incidence dropped to the level in line with the increasing incidence observed before screening started. The pattern for Fyn was similar to that of Copenhagen for the first invitation round with a pronounced prevalence peak, and the incidence decreased during the second invitation rounds but seemed a little higher than before screening started. The picture for the small municipality of Frederiksberg was different, as the incidence continued to be high during the second invitation round. The incidence of breast cancer for the rest of Denmark increased slightly during the years 1985–1997.

## DISCUSSION

### Clinical mammography/opportunistic screening and background incidence

Opportunistic screening has not been widespread in Denmark. Even in 2000 only 59 000 clinical mammograms were performed in a population of 5.3 million compared with, for example, 220 000 clinical mammograms in Norway in the mid-1990s in a population of 4.5 million (Wang, 2002). In 2000, 25 000 clinical mammograms were undertaken in the 500 000 women aged 50–69, whereas there were 38 000 screening mammograms in 100 000 such women in the three screening programmes.

It is, therefore, reasonable to assume that the incidence of invasive breast cancer among women aged 50–69 in the rest of Denmark during the period 1985–1997 represented the incidence in a population with low-intensity opportunistic screening. Incidence was quite stable at these ages from the start of the Danish Cancer Register in 1943 until 1960, after which the incidence increased by more than 50% to the mid-1980s (Sundhedsstyrelsen, 2001). Our analysis showed that this increase continued in the rest of Denmark in the late 1980s and 1990s.

### Expected incidence of invasive breast cancers after the start of screening

In a closed, nonageing population, the introduction of mammography screening that detects early invasive cancers with a 100% sensitivity and with a 100% participation rate would be expected to result in an increase in the incidence of invasive cancer during the first invitation round, as prevalent asymptomatic lesions are detected. When this population is rescreened at regular intervals, incidence of invasive cancer is expected to drop to the prescreening level, if the screening does not lead to overdiagnosis of cases that would otherwise not have given rise to clinical symptoms.

In an open, nonageing population with a screening programme that detects early invasive cancers with a sensitivity below 100%, with a participation rate below 100%, and offered biennially to women aged 50–69, it is difficult to predict the level to which incidence will drop after the first round, as there are forces working in opposite directions. Any new invitation round will be a prevalence round for newcomers to the programme, i.e. women turning 50, women moving into the screening area, and previous nonparticipants, and this will contribute to pushing the incidence above the prescreening level. The lower sensitivity implies that some prevalent cases will be left for detection at the second or third screen. A certain proportion of screen-detected cases will also be left for assessment and final diagnosis during the period of the next invitation round. A decline in the participation rate over invitation rounds will, on the other hand, tend to lower the incidence.

If screening also detects ductal carcinoma *in situ* (DCIS) cases, the incidence of invasive cancer is expected eventually to become lower in the rescreening rounds than before screening. If there is an underlying increase in the background risk of breast cancer, these screening-induced changes in incidence will be added to the background trend.

### Observed incidence of invasive breast cancer after start of screening

In both Copenhagen and Fyn, a prevalence peak was clearly shown, followed by an incidence during subsequent rounds fairly similar to that expected without screening. Compared to the prescreening period, the incidence was roughly doubled in the prevalence round in both regions. Given no overdiagnosis, this roughly implies that, on average, screening brings forward diagnosis by about 2 years. The mean sojourn time in mammography screening has been estimated as about 4 years in the age group 50–69 (Chen *et al*, 1997). An average lead time of about 2 years would therefore not be an unreasonable estimate. Therefore, the magnitude of the prevalence peak in Copenhagen and Fyn can be interpreted as an indication of lead time and thus of no overdiagnosis in the prevalence round.

In both regions, about 18% of the participants in the second invitation round were newcomers. The sensitivity for detection of invasive cancers was calculated as screen detected cancers/(screen detected cancers + interval cancers). During the first invitation round, sensitivity was 87% in Copenhagen and 80% in Fyn. In Copenhagen, 11% of the invasive cases detected during the first invitation round had the final diagnosis made during the second invitation round, and in the present analysis were counted as incident cases during the second round. In Copenhagen, the participation rate declined from 71% during the first invitation round, to 65 and 63% in the subsequent rounds, tending to lower the observed incidence during the later rounds. In Fyn, 88% participated in the first round. Thus, several factors tended to push the incidence upwards during the second and third invitation rounds, and only one factor operated in the opposite direction. This incidence was similar to that expected without screening and therefore seems a good indicator of no overdiagnosis.

In both Copenhagen and Fyn, throughout the programme about 11% of the screen-detected cases were DCIS. We tabulated the combined incidence of invasive breast cancer and screen-detected DCIS cases, but the pattern was almost identical with that for invasive cancer only (data not shown).

In Frederiksberg, the increase in the incidence continued after the first invitation round. This small municipality is located within Copenhagen, and the incidence prior to screening was similar in the two areas. It should be noted that the second invitation round was postponed for 4 months when Frederiksberg was incorporated into the Copenhagen programme. The detection rate of invasive cancer and DCIS was 9.5 per 1000 screened in the first invitation round in Frederiksberg compared with 11.9 per 1000 screened in Copenhagen. In the second invitation round, however, the detection rate in Frederiksberg was 8.2 per 1000 screened, whereas it was 6.3 per 1000 in Copenhagen. The two municipalities thus had similar *average* detection rates for the first two invitation rounds: (9.5+8.2)/2=8.9 and (11.9+6.3)/2=9.1, respectively.

Our study showed that in Copenhagen and Fyn, incidence during the second and third invitation rounds was fairly similar to that expected in the absence of the screening. It is therefore reasonable to conclude that screening did not lead to overdiagnosis of breast cancer. The pattern was different in the small municipality of Frederiksberg, where the continued high incidence during the second invitation round was probably partly because of a low sensitivity during the first round.

Overdiagnosis is an important potential disadvantage of screening, which is difficult to monitor in gradually introduced or opportunistic screening. Our results indicate that well-organised mammography screening can operate without overdiagnosis of breast cancer.
